# Supplementary material for: COVID-19 Vaccinations, Trust, and Vaccination Decisions within the Refugee Community of Calgary, Canada
Source: Vaccines (Basel). 2024 Feb 9;12(2):177. doi: 10.3390/vaccines12020177 (PMC10891815; doi:10.3390/vaccines12020177)
Supplement: Supplementary file 1 [file vaccines-12-00177-s001.zip › vaccines-2841356-supplementary.pdf]

Table S1. Determinants of under vaccination among migrant populations.

Determinants and Characteristics
<u>Information inequities</u><br>● Limited access to health information leading to inadequate knowledge about vaccinations and vaccine-preventable diseases [6-7].<br>● Language barriers and/or low literacy [4,6-8].<br>● Vaccine misinformation [3-4,9].<br>● Low health literacy [4,9].<br>● Information not accessible or not provided in an acceptable format [7].<br>● Inadequacy of public health vaccination campaigns targeting migrant groups [10].<br>● Confusion related to vaccine eligibility [11].<br><u>Personal beliefs</u><br>● Low risk perception of vaccine-preventable disease [8].<br>● Vaccine-specific hesitancy (e.g. fears of adverse effects, vaccine safety concerns) [1,7, 2].<br>● Ethnic, cultural, or religious values [1,6-7,12].<br><u>Previous systems experiences</u><br>● Experiences of racism, discrimination, or social exclusion within health systems [1,4,7,9,13].<br>● Distrust of the health/medical system and/or government systems [4,6,9].<br>● Fear of accessing the healthcare system based on legal status such as undocumented migrants [11,14].<br><u>Structural inadequacies</u><br>● Inadequate vaccine delivery coordination by public health authorities [7, 14].<br>● Gaps in national policies on migrant/refugee health services and vaccinations [1, 7, 13].<br>● Lack of vaccine coverage in the country of origin [14].<br>● Barriers or inequities to accessing healthcare in general translate to vaccine access [1,13].<br><u>Accessibility of vaccine services</u><br>● Lack of awareness and/or physical access to immunization services [7].<br>● Difficulties navigating the healthcare system [1,13].<br>● Poor digital literacy and lack of access to technology for booking and accessing information [7].<br>● Financial barriers accessing vaccination; including indirect costs such as taking time off work [1,5,7,13].<br><u>Intersecting risk factors for undervaccination</u><br>● Level of education [8].<br>● Income [1, 13].<br>● Family size, birth order of child [7].<br>● Age [15].<br>● Country/continent of origin [15].<br>● Geographic location (e.g., rural/urban) [15].<br>● Faith and/or culture [1, 6-7,12].<br>● Legal status [14].
